# Cruelty in the Crib

**Published:** 1888-05

**Authors:** 


### CRUELTY IN THE CRIB.

We infer from the following realistic picture, that our bachelor friend the Editor in chief of *Thought of the Times*, from which we extract it, has a remarkably good memory :

" Superintendent Charidi, of the Society for the Prevention of Cruelty to Children, would have his hands full if complaints could come from the cradle. The baby's torture begins directly after birth. It is soaped and soaked, sopped and scrubbed, wiped and wobbled and walloped, all to get it clean. Then it is bound up in strong bandages so that it will not burst in crying. The bandage about the loins is pulled as tightly as possible and pinned. Swaddling napkins, flannel underclothes and skirts are then girted around it, and pinned as tightly as they can be pulled. The head is combed and curried and smeared with some sort of ointment, and then the little sufferer is wound up in woolen wraps and laid away. It is so bound up and wrapped up and covered up, that it can scarcely get breath to squall. Pins prick and it kicks with pain. Then come the paregoric and soothing syrup, castor oil molasses and whiskey. It is rubbed and rocked, trotted and tossed, and dosed with drug stuff to cure the supposed colic. If there be no one about with any common sense, the chances are good for a child's funeral."

